# Efficacy of Different Root Canal Irrigating Solutions in Removing Smear Layer: A Scanning Electron Microscopic Study

**DOI:** 10.7759/cureus.44618

**Published:** 2023-09-03

**Authors:** Zinnie Nanda, Romalpreet Singh, Priyanka P Kamble, Gargi Deshmukh, Nileshrao Patil, Anshuman B Patil, Satyabrat Banerjee

**Affiliations:** 1 Conservative Dentistry and Endodontics, Jawahar Medical Foundation's (JMF) Annasaheb Chudaman Patil Memorial (ACPM) Dental College, Dhule, IND; 2 Conservative Dentistry and Endodontics, Desh Bhagat Dental College, Malout, IND; 3 Oral Pathology and Microbiology, Jawahar Medical Foundation's (JMF) Annasaheb Chudaman Patil Memorial (ACPM) Dental College, Dhule, IND; 4 Dental Surgery, Chhatrapati Shahu Maharaj Shikshan Sanstha's (CSMSS) Dental College and Hospital, Aurangabad, IND

**Keywords:** ethylene diamine tetraacetic acid, scanning electron microscopy, smear layer, sodium hypochlorite, root canal irrigants

## Abstract

Background

The aim of the present investigation was to evaluate and compare the efficacy of frequently used chemical agents in terms of their capacity to eliminate the smear layer after instrumentation, as observed via scanning electron microscopy (SEM).

Materials and methods

Sixty extracted single-rooted mandibular premolar teeth, each with roots 15 mm in length, were used in this study. The teeth were divided into one control group and four study groups, each containing 12 teeth. In Control Group 0, teeth were irrigated with 3 ml of saline only. In Group 1, teeth were irrigated initially with 3% sodium hypochlorite (NaOCl) and then given a final rinse with 3 ml of 17% ethylenediaminetetraacetic acid (EDTA) for one minute. In Group 2, teeth were irrigated with 3% NaOCl and given a final rinse with 3 ml of a mixture of tetracycline, acid, and detergent (MTAD, BIOPURE) for one minute. In Group 3, teeth were irrigated with saline and given a final rinse with 3 ml of 17% EDTA for one minute. In Group 4, teeth were irrigated with saline and given a final rinse with 3 ml of MTAD for one minute. One-half of each tooth was chosen and prepared for scanning electron microscopic (SEM) examination at the cervical, middle, and apical thirds. These were observed at magnifications of up to 1,000 times to check for the presence or absence of a smear layer. The data were analyzed using the Kruskal-Wallis test and post-hoc Dunn's test.

Results

All of the root canal irrigation protocols exhibited superior efficacy compared to the control group in the elimination of the smear layer. Group 2 (3% NaOCl with MTAD) showed the lowest mean scores, compared to all the groups, followed by Group 1 (3% NaOCl with 17% EDTA). MTAD was more effective than EDTA. The smear layer was effectively removed from the apical third, followed by the middle and coronal thirds of the root.

Conclusion

Initial irrigation with 3% NaOCl and one-minute final irrigation with 3 ml MTAD was the most effective root irrigant, and particularly indicated in teeth with infection of the apical third.

## Introduction

The treatment of endodontic diseases is primarily based on the elimination of calcified material through mechanical means and the chemical sterilization and dissolution of organic debridement within the intricate architecture of the root canal [1]. The irrigation procedure holds a crucial position in the entire process as it substantially contributes to the eradication of microorganisms present within the complex root canal structure. Diverse irrigation solutions have been extensively studied at varying concentrations, either individually or in conjunction with sodium hypochlorite, ethylenediaminetetraacetic acid (EDTA), citric acid, tetracycline, and chlorhexidine [2,3].

Multiple investigative studies that utilized scanning electron microscopy (SEM) have shown the efficacy of sodium hypochlorite (NaOCl) irrigation in debris removal [4]. However, one should note that there is a significant disadvantage associated with NaOCl: its potential cytotoxic effects when inadvertently injected into peri-radicular tissue. Despite NaOCl's heightened efficacy as an irrigation agent, it has limited ability to dissolve the inorganic components of the smear layer. The smear layer, a byproduct of dentin removal, encases the entire wall of the root canal [5] and is not easily dissolved by NaOCl. Scelza et al. suggested that this smear layer could serve as a repository for bacteria or their byproducts, thereby acting as a reservoir for irritants [6].

The use of chelating agents, such as EDTA, during the biomechanical preparation of root canals helps in the elimination of the smear layer. This, in turn, enhances the penetration of irrigants into the dentinal tubules [7]. Such increased permeability contributes to comprehensive disinfection of the root canals. The elimination of the smear layer could also improve the accessibility of dentinal tubules, boost the efficacy of intracanal medication, and facilitate better bonding of the root canal-filling material.

Conventional irrigation protocols involve sequential applications of NaOCl in concentrations ranging from 0.5% to 5.25% and 17% EDTA [8]. Therefore, the objectives of endodontic treatment go beyond just the removal of pulp residue and canal enlargement; there is also a need for focused attention on the eradication of the smear layer. The integration of chelating agents into NaOCl solutions reduces pH in a manner that is both ratio- and time-dependent. The significant decrease in available chlorine in NaOCl blends, due to chemical interactions with EDTA, appears to account for the incapability of NaOCl and EDTA mixtures to solubilize soft tissues [2].

MTAD, a composite mixture that includes the tetracycline isomer, an acidic component, and a detergent, is broadly accepted as an effective irrigant with antibacterial properties. It is mainly used as a final rinse during root canal cleaning. This particular solution has a crucial and indispensable role in removing the smear layer that exists on the inner surface of a treated permanent root canal. Moreover, with its acidic pH of 2.15, this solution shows a strong ability to remove inorganic matter from the root canal while also preserving the structural integrity of the dentinal tubules [9].

The use of MTAD in combination with NaOCl has not been widely studied, leading to a lack of research data. To address this important gap, the current study was initiated to examine the efficacy of different final irrigating solutions in the removal of the smear layer, specifically 17% EDTA or MTAD with or without a follow-up 3% NaOCl irrigation. This study aims to test the null hypothesis that there is no noticeable difference in the efficacy of various irrigants for removing the smear layer.

## Materials and methods

Sample selection

The study was approved by the institutional ethics committee of Jawahar Medical Foundation's (JMF) Annasaheb Chudaman Patil Memorial (ACPM) Dental College (EC/NEW/INST/2022/2959/198). Sixty human single-rooted mandibular premolars, which were recently extracted for orthodontic or periodontal purposes, were procured for the investigation.

Inclusion and exclusion criteria

For this study, only teeth exhibiting fully developed roots with no open apices were selected, while those with caries, fractures, or signs of resorption were deliberately excluded. Furthermore, teeth with dilacerated roots, multiple root canals, or fused root canals were excluded from consideration.

Method of collection and storage of teeth

Extracted human premolar teeth were collected, stored, disinfected, and handled as per the recommendation and the guidelines laid down by the Occupational Safety and Health Administration (OSHA) and the Centers for Disease Control and Prevention (CDC).

Teeth preparation and allocation of groups

All specimens' crowns were removed at the coronal portion of the tooth to achieve a uniform working length of 15 mm, using diamond discs for the purpose. After decoronation, the teeth were divided into five distinct groups, with each group containing 12 teeth. This division aimed to facilitate a more organized and structured analysis of the samples. Access openings were made, and working lengths were set using a K-Type #10 file (Dentsply) until visible at the apical foramen with an Endo Gauge (Dentsply). Following the manufacturer's guidelines, the crown-down technique was used, and all teeth in each group were instrumented with the ProTaper Universal System (Dentsply). The canals were outfitted consecutively with ProTapers of 21 mm length in varying sizes, specifically Sx, S1, S2, F1, and F2, to ensure each canal reached a resistance level of 300 rpm in the electric torque control motor.

Subsequently, each sample was irrigated with 3 mL of the designated irrigant for exactly 60 seconds. The application of irrigants into the root canal adhered strictly to the protocols defined for each group, using a double-vented 30-gauge endodontic irrigation needle.

The groups were divided as follows according to the treatment administered: Control Group 0 involved root canals irrigated with 3 mL saline only; Group 1 featured root canals irrigated with 3% NaOCl during mechanical preparation, followed by a final rinse with 3 mL 17% EDTA for one minute; Group 2 consisted of root canals irrigated with 3% NaOCl between mechanical preparations, and then given a final rinse with 3 mL of MTAD for one minute; Group 3 had root canals subjected to saline irrigation between mechanical preparation, followed by a final rinse with 3 mL of 17% EDTA for one minute; and Group 4 included root canals irrigated with saline between mechanical preparation, with a final rinse of 3 mL of MTAD for one minute.

SEM analysis

In order to create deep grooves without puncturing the buccal and lingual surfaces of the root, diamond disks were used. The roots were carefully split into two halves with the aid of a chisel and surgical mallets. One of the split sections for each specimen was randomly selected for SEM analysis (JSM-6390, JEOL, Japan) at 1000x magnification for the presence or absence of a smear layer. The cervical, middle, and apical thirds were analyzed, providing three sections from each portion for SEM analysis.

Scoring criteria

Scoring criteria were according to the rating system developed by Rome et al. (Table 1) [10].

**Table 1:** Scoring criteria for smear layer assessment given by Rome et al.

Scoring	Description
0	No smear layer, dentinal tubules open, free of debris
1	Root canal surfaces with residue only at the opening of the dentinal tubules
2	Root canal surfaces with a thin covering of residue on dentinal tubules with visible tubules only in a few regions
3	Heavy smear layer, outlines of dentinal tubules totally covered

The scoring process was performed by two separate observers who were blinded to any potential biases that could have affected their judgments. Subsequently, the resulting scores were meticulously assessed and scrutinized, and the average of these scores was ultimately deemed the final, definitive measurement.

Statistical analysis

The inter-examiner reliability was assessed using the kappa test. For comparing the mean scores across groups, non-parametric tests like the Kruskal-Wallis test were utilized, followed by post-hoc analysis using Dunn's test. All statistical analyses were carried out at a significance level of p < 0.05.

## Results

**Figure 1:**
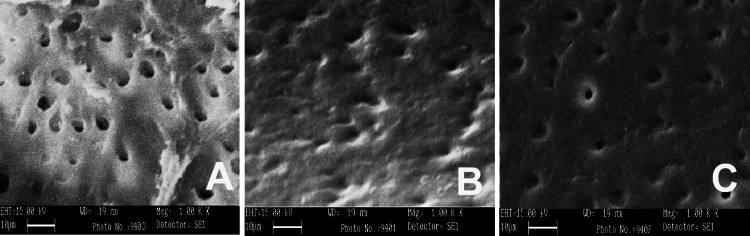
SEM images of Group 0 from apical (A), middle (B), and coronal (C) areas irrigated with saline only. SEM: scanning electron microscopy.

**Figure 2:**
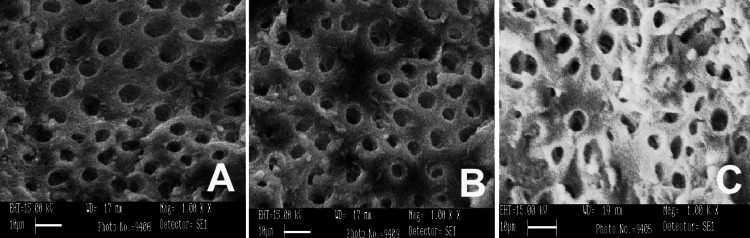
SEM images of Group 1 from apical (A), middle (B), and coronal (C) areas irrigated with 3% NaOCl and 17% EDTA. SEM: scanning electron microscopy; EDTA: ethylenediaminetetraacetic acid.

**Figure 3:**
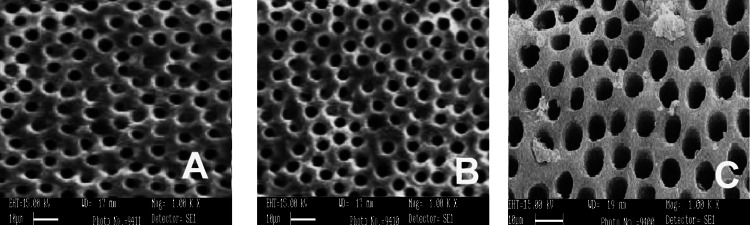
SEM images of Group 2 from apical (A), middle (B), and coronal (C) areas irrigated with 3% NaOCl and MTAD. SEM: scanning electron microscopy; MTAD: mixture of tetracycline, acid, and detergent.

**Figure 4:**
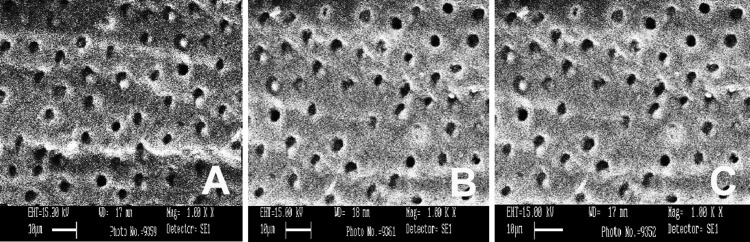
SEM images of Group 3 from apical (A), middle (B), and coronal (C) areas irrigated with saline and EDTA. SEM: scanning electron microscopy; EDTA: ethylenediaminetetraacetic acid.

**Figure 5:**
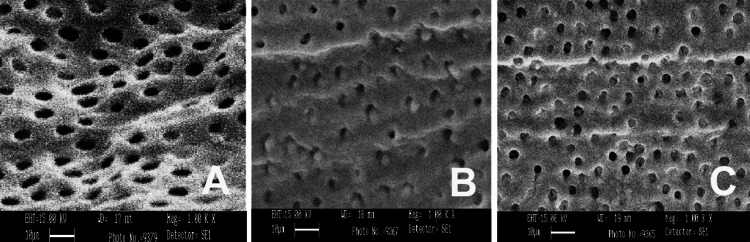
SEM images of Group 4 from apical (A), middle (B), and coronal (C) areas irrigated with saline and MTAD. SEM: scanning electron microscopy; MTAD: mixture of tetracycline, acid, and detergent.

SEM was conducted using three different irrigants: 3% NaOCl, 17% EDTA, and MTAD. SEM images were captured from the apical, middle, and coronal sections of the samples at a magnification of 1000×, as shown in Figures 1-5. As revealed by the SEM images of Group 0, saline was the least effective in removing the smear layer across all regions of the root canal, a finding that was supported by statistical analysis. The SEM images from Group 1, which used 3% NaOCl and 17% EDTA, displayed remnants of smear at the openings of the dentinal tubules. In contrast, Group 2, employing 3% NaOCl and MTAD, showed clear openings of dentinal tubules in the apical, middle, and coronal sections. For Groups 3 and 4, the intratubular smear was more prominently visible in the middle section compared to the apical and coronal areas.

 

In assessing inter-examiner reproducibility, both observers showed an exceptional level of agreement, as reflected by observed kappa values ranging from 0.88 to 0.96. These results not only underscore the reliability of the evaluation methodology used in this study but also suggest that the findings may be generalizable to broader patient populations.

The null hypothesis was rejected in this study. Table 2 displays statistically significant differences in mean score values across all groups (p < 0.05). The lowest mean score was recorded for Group 2 (1.08), followed by Groups 1, 4, and 3. The control group exhibited the highest scores. According to these findings, initial irrigation with 3% NaOCl, followed by a final rinse with MTAD, was most effective in smear layer removal. Even MTAD alone yielded better outcomes than did 17% EDTA and saline (Group 3), or irrigation with only saline (control group).

**Table 2:** Descriptive analysis of mean scores of smear removal by different irrigation solutions. *p-value < 0.05: significant.

Groups	Size (n)	Mean score	Standard deviation	Median	Min	Max	Kruskal-Wallis	p-value
Group 0	12	2.75	0.45	3	2	3	37.319	<0.001*
Group 1	12	1.50	0.52	1.5	1	2		
Group 2	12	1.08	0.67	1	0	2		
Group 3	12	2.42	0.67	2.5	1	3		
Group 4	12	2.25	0.75	2	1	3		

Table 3 shows that Group 2, in which the irrigation was performed with initial rinse of 2.5 % NaOCl, followed by a final rinse with MTAD, was most effective in removing the smear layer from the apical, middle, and coronal regions, followed by Groups 4 and 3. The control group (irrigation with saline only) was ineffective in removing the smear layer and performed better with the final rinse with EDTA or MTAD. MTAD performed better than EDTA at removing the smear layer. All the root canal irrigants were effective in removing smear layer from apical region of the teeth, compared to middle and coronal regions.

**Table 3:** Mean scores of smear removal in different regional areas of root canal.

Groups	Size (n)	Apical (mean score±SD)	Middle (mean score±SD)	Coronal (mean score±SD)
Group 0	12	2.67±0.43	2.98±0.86	2.42±1.02
Group 1	12	1.28±0.29	1.72±0.43	1.52±0.42
Group 2	12	0.97±0.18	1.21±0.64	1.07±0.45
Group 3	12	2.12±0.76	2.72±0.98	2.16±0.97
Group 4	12	2.01±0.39	2.67±0.94	2.09±0.98

Table 4, based on post-hoc analysis, indicates that Groups 2, 3, and 4 had statistically significant differences compared to the control group. Meanwhile, Group 1 displayed non-significant differences (p > 0.05). These findings suggest that the use of 3% NaOCl alone as an irrigant is not effective in removing the smear layer. This is in contrast to its use in combination with 17% EDTA or 3 ml of MTAD, where it performed significantly better. In this context, 3% NaOCl behaved almost similarly to normal saline.

**Table 4:** Post-hoc comparison of groups by Dunn's test. *p-value < 0.05: significant.

Groups	Difference	Statistic	p-level
Group 1			
vs Group 2	0.417	2.588	p<0.05*
vs Group 3	0.917	0.474	p>0.05
vs Group 4	0.75	3.616	p<0.05*
vs Group 0	1.833	2.075	p>0.05
Group 2			
vs Group 3	1.333	2.114	p>0.05
vs Group 4	1.167	1.028	p>0.05
vs Group 0	2.250	4.430	p<0.05*
Group 3			
vs Group 4	0.167	3.142	p<0.05*
vs Group 0	0.917	2.501	p<0.05*
Group 4			
vs Group 0	1.083	5.254	p<0.05*

## Discussion

One of the crucial and significant aims of endodontic therapy is to perform debridement of the root canals. This ultimately results in the complete removal of pulp tissue, necrotic debris, microorganisms, and their toxins. Root canal irrigants play a fundamental role in the chemo-mechanical preparation process by effectively eliminating and eradicating the by-products formed during the aforementioned procedure [11]. The results of this study demonstrated that all irrigants were significantly effective in removing the smear layer compared to saline irrigation. These findings were in accordance with previous studies [8,9,11,12].

In the current investigation, irrigation solutions such as 3% NaOCl, 17% EDTA, and MTAD were used, with saline water serving as a control. Three percent NaOCl was chosen for this study because higher concentrations of 6-8.5% have known cytotoxic effects. Lower concentrations between 0.5 and 2% show less bactericidal properties [13]. Our study noticed that the use of 3% NaOCl was effective in removing the smear layer compared to irrigation with only saline. Sodium hypochlorite demonstrates characteristics of both oxidizing and hydrolyzing agents. Owing to its strong proteolytic effect, it serves as an exceptional aid during instrumentation. It is widely recognized for its ability to break down and degrade proteinaceous matter effectively. Hypochlorous acid, a chemical species found in sodium hypochlorite solutions, exhibits solvent-like behavior upon encountering organic tissue. It liberates chlorine, which then engages in a chemical reaction with the amino group of the constituent protein, ultimately resulting in the formation of chloramines that interfere with cellular metabolism.

The smear layer, an amalgamation of both mineralized and organic components, consists of fragments of odontoblastic processes and necrotic debris. It has the potential to serve as a habitat for bacteria. Moreover, it impedes the sealing efficiency of root canal filling, thereby acting as a physical hindrance. To circumvent these detrimental consequences, practitioners often turn to irrigation to remove the smear layer. This process requires the use of a chelating agent and a soft tissue solvent. One effective irrigation procedure that has been suggested involves the use of a combination of EDTA and NaOCl. This has been demonstrated to thoroughly disinfect the root canal and eliminate both organic and inorganic materials. EDTA is a potent chelating agent capable of effectively removing the smear layer from dental hard tissues [12]. Recent research has suggested that EDTA also possesses remarkable antimicrobial properties. Dentin can be decalcified to an astonishing depth of 20-30 μm within a five-minute duration [14]. Saito et al. reported that the group receiving one minute of EDTA irrigation demonstrated significantly higher removal of the smear layer than groups that received either 30 or 15 seconds of EDTA irrigation [15]. Nonetheless, the use of EDTA in conjunction with NaOCl has its drawbacks. It has been observed to diminish the microhardness of dentin and alter its flexural strength and modulus of elasticity, resulting in irreparable damage to the dentin's microstructure and making teeth more susceptible to fracture [12].

Torabinejad et al. devised an irrigant that combines chelating and antibacterial properties. This concoction, known as MTAD, comprises a blend of 3% doxycycline, 4.25% citric acid, and detergent (Tween-80) [16]. Citric acid, a crystalline organic acid with antibacterial properties, facilitates the removal of smear layers. It enhances the capacity for doxycycline to penetrate deeper into the dentinal tubules, thus exerting its antibacterial effects. The inclusion of Tween 80, a nonionic surfactant and detergent, in MTAD results in a reduction of the surface tension of saline water and NaOCl, facilitating an increase in the flow and penetration of irrigating solutions into the dentinal tubules. This ultimately leads to complete disinfection of the canal spaces [17].

Our study demonstrated that the use of MTAD with NaOCl was more effective in removing the smear layer compared to MTAD alone. This finding aligns with those of Torabinejad et al., who concluded that MTAD demonstrated a superior capacity for removing the smear layer compared to various concentrations of sodium hypochlorite [16]. The effectiveness of MTAD with NaOCl was most notable in the apical third of the root canal. Therefore, it can be used in teeth with infection in the apical part. This was in accordance with Kumar et al. [18].

In the current study, EDTA was less effective than MTAD, in line with previous studies [1,5,7]. This could be because when EDTA is used with NaOCl, it reduces the amount of free chlorine release, thus decreasing its ability to dissolve soft debris. Similar findings were reported by Irala et al. [19]. Another study indicated that NaOCl had little effect on the calcium-chelating ability of EDTA [20]. In contrast, the pH value of MTAD was recorded to be 2.15, contributing to its function as a calcium chelator and leading to the demineralization of the enamel and root surface. This effectively removes the smear layer [21]. MTAD has also been found to be minimally cytotoxic, with no adverse effects on the physical properties of dentin, unlike EDTA [22].

Most conducted studies aiming to compare the efficacy of root canal irrigants have used EDTA and MTAD in combination with 5.25% NaOCl [9,11,22]. However, due to concerns about cytotoxicity associated with the use of NaOCl, the current study investigated the efficacy of MTAD and EDTA as sole irrigants. The results have shown that these irrigants effectively eliminate the smear layer from all areas of the root canal. Low concentrations of NaOCl were used in our study, proving to be effective when used in conjunction with MTAD and resulting in minimal cytotoxicity compared to the 5.25% NaOCl employed in previous studies [9,11,12,20-22].

Clinical implications of the study

Root canal irrigation with 3% NaOCl initially, and then a final rinse with 3 ml of MTAD for one minute can be effectively used to remove the smear layer, without affecting the physical properties of dentin, and thus is safe to use.

Limitations

The present study did not evaluate the effects of different irrigants on the physical properties of dentin or on the microbial count. Future prospective, in vivo studies are imperative to further investigate the efficiency of MTAD and other irrigation solutions as intracanal irrigants.

## Conclusions

Within the confines of the current in vitro investigation and the use of SEM for data analysis, it can be concluded that a finishing irrigation with 3 ml of MTAD for one minute, following initial rinsing with 3% NaOCl, was the most effective irrigation regimen compared to 17% EDTA. The use of intracanal irrigants proved better for eliminating the smear layer than did normal saline irrigation. Future research on irrigants requires concentrated effort to discover a single irrigant capable of dissolving tissue, removing smear deposits, and exhibiting antibacterial properties.
